# *Finding My Way* from clinical trial to open access dissemination: comparison of uptake, adherence, and psychosocial outcomes of an online program for cancer-related distress

**DOI:** 10.1007/s00520-022-07205-0

**Published:** 2022-06-22

**Authors:** Lisa Beatty, Emma Kemp, Bogda Koczwara

**Affiliations:** 1 College of Education, Psychology & Social Work, Flinders University, GPO Box 2100, Adelaide, SA 5001 Australia; 2 College of Medicine & Public Health, Flinders University, Adelaide, Australia

**Keywords:** Digital health, Psycho-oncology, Intervention, Dissemination, Open access

## Abstract

**Purpose:**

Few digital psycho-oncology programs have been adopted into routine practice; how these programs are used after trial completion remains unexplored. To address this, the present study transitioned our evidence-based 6-module CBT-based program, *Finding My Way*, into open access (OA) after completion of the RCT, and compared uptake, usage, and psychosocial outcomes to the earlier RCT.

**Methods:**

Recruitment was passive, via promotion through (1) media and social media releases, (2) public lectures, (3) radio interviews and podcasts, and (4) clinician-initiated referral. Measures included number of enrolled users, number of modules completed, and pre- and optional post-measures of distress and quality of life (QOL).

**Results:**

Uptake was lower in OA (*n* = 120; 63% of RCT). Usage was markedly lower: 1.5 modules were completed on average (vs 3.7 in RCT), and only 13% completed a ‘therapeutic dose’ of 4 + modules (vs. 50% in RCT). Research attrition was high; *n* = 13 completed post-measures. OA users were more sociodemographically and clinically diverse than RCT users, had higher baseline distress (OA *M*_*pre*_ = 36.7, SD = 26.5; RCT *M*_*pre*_ = 26.5, SD = 21.7), and reported larger pre-post reductions than their RCT counterparts (OA *M*_*post*_ = 23.9, SD = 20.7; RCT *M*_*post*_ = 21.2, SD = 21.2). Moderate improvements in mental QOL occurred during OA (*M*_*pre*_ = 37.3, SD = 12.6; *M*_*post*_ = 44.5, SD = 12.1), broadly replicating RCT findings.

**Conclusion:**

Findings that OA users were more medically and sociodemographically diverse and distressed at baseline than their RCT counterparts, and — despite having lower usage of the program — achieved larger changes from baseline to post-program, will help to shape future intervention design, tailoring, and dissemination.

## Introduction

While the empirical evidence base for digitally delivered psychosocial programs for those affected by cancer is rapidly growing [1–7], a research-practice gap has emerged, with few interventions subsequently adopted into routine oncology care [8, 9].

Two recent systematic reviews explored facilitators and barriers to implementing web-based therapy and digital health tools, from the perspectives of (a) researchers/program developers [8] and (b) health care providers (HCPs)/referrers [9]. The first review identified 26 efficacious self-directed digital health tools that have been trialled in cancer, of which only six (27%), were disseminated to end-users after the trial [8]. The main barriers to dissemination were limited funds, lack of infrastructure, and limited research timelines [8]. The second review identified that HCPs would only support web-based therapies for patients with (a) relatively straightforward/low-risk diagnoses, (b) strong motivation, (c) high computer literacy and access, and (d) low need for tailored content [9]. The review authors concluded that to increase uptake in routine care, it would be important to ensure HCPs receive education and training to support them in referring to, and incorporating, these therapies into practice. However, our group recently found that even when HCPs were familiar with two locally developed Australian programs, referral barriers continued [10]. More specifically, there was a lack of trust in online program content and processes, and a belief that optimal patient outcomes would be better achieved via a blended model of care where a HCP delivers tailored therapy combined with online components where appropriate [10].

These findings suggest that there are barriers to both (i) making programs available post-RCT, and (ii) encouraging HCPs to refer to programs where they *are* available. However, should these barriers be overcome, a third challenge exists that has remained relatively unexplored and unreported: of those programs that have been disseminated to end users after clinical trial completion, to what extent are they adopted and used by end-users (e.g., cancer patients and survivors)? This information is key to informing whether digital mental health programs are worth the substantial resource and training investments that are required to develop, test, and disseminate these interventions. Only one study to date has reported on the clinical practice implementation of their online transdiagnostic clinician-monitored CBT program for cancer-related depression and anxiety [11], finding that only 2% of eligible/screened patients were referred to the *iCanAdapt* program by clinicians, only 25 patients self-referred over the 12 month recruitment window, and only 44% completed 1 or more modules. However, as *iCanAdapt* was offered as a component of a broader clinical treatment pathway, rather than as a standalone program, how well these findings translate to uptake and usage when offered/promoted in isolation remains unknown.

Following the successful completion of the RCT of our digital psycho-oncology program, *Finding My Way* — which found improved emotional quality of life and reduced health service usage over time for intervention users compared to a psychoeducational control group [1] — our group was able to make the program freely available to the oncology community. We therefore sought to address the knowledge gap, by conducting an open access study over a matched recruitment timeframe to the RCT. The aim of the present study was to compare uptake, usage, and psychosocial outcomes between our ‘real world’ open access and RCT cohorts.

## Methods

### Setting and design

The *Finding My Way* (FMW) clinical trial protocol has been published elsewhere [12]. The present study was a single-group pre-post open access trial. Participation in the study occurred entirely online via www.findingmyway.org.au. Ethics approval was obtained from the Southern Adelaide Clinical Human Research Ethics Committee (476.16).

### Participants

*FMW* is aimed at adult (aged over 18 years) acute cancer survivors, defined as those diagnosed in the past 6 months with any cancer treated with curative intent and currently receiving anti-cancer treatment. Participants had to have sufficient English proficiency to be able to register/consent for, and utilise, the program, and have access to the internet and an active email address. During open access, as recruitment occurred via self-selection, eligibility required self-screening and self-selecting.

### Procedure

Participants in the current study were recruited over a 2-year window (16/2/2017–14/2/2019) to match the RCT’s 2-year recruitment timeframe. Recruitment methods were passive, including media releases, social media advertising through professional networks (Cancer Council SA; Flinders University); radio interviews; podcasts; and clinician-initiated referral. Given there were no study coordinators or direct approach within clinics, nor recruitment reminders to referrers, any referrals were due to clinicians’ own awareness of the program, and desire to refer to it.

Eligible participants completed an initial online registration, where they created their username and password, followed by a brief demographic and psychosocial questionnaire. Following this, they were directed to a tutorial on their personalised user homepage instructing them how to use the program. Six weeks later, participants received an email link to complete an optional follow-up assessment post-intervention, with an automated email reminder 1 and 2 weeks later.

### Intervention

*FMW* has been described in detail elsewhere [1, 12]. The program is a 6-week/6 module password-protected web-based resource comprising (i) psychoeducation, in written and video formats; (ii) cognitive behaviour therapy-based strategies (including worksheets, quizzes, and relaxation/meditation exercises; and (iii) survivor testimonials, in video and written formats. The 6 modules, released at a rate of one per week, address common psychosocial concerns following diagnosis, including (a) navigating *diagnosis,* starting treatment and communicating with the treatment team; (b) coping with *physical symptoms* and side effects; (c) managing *emotional distress*; (d) coping with changes in *how you see yourself* (identity, body image and sexuality); (e) concerns with *your family and friends*, and (f) issues arising as participants contemplate *completing treatment* and transitioning to post-treatment survivorship. Upon first access to the program, participants could self-tailor/rearrange the order of modules. A booster module, summarising key program strategies, became accessible one month after program completion. Participants had ongoing access to all program materials after all modules and assessments were released.

### Measures

#### Demographic and clinical characteristics

Characteristics recorded at baseline included sex, age, marital status, employment status, level of educational attainment, annual gross income, cultural affiliation, cancer type, date of diagnosis, and treatments received (surgery, chemotherapy, radiotherapy, other adjuvant treatments). One item ‘in what capacity are you using the program’ was added to the open access survey mid-way through data-collection, when it became clear that users without cancer were enrolling into the program (i.e., health care professionals, and family-members/carers).

#### Uptake and engagement

Uptake was assessed via number enrolled participants within a matched recruitment timeframe to the RCT, and engagement/usage was assessed via number of modules completed (range 0–6).

#### Effectiveness

Intervention effectiveness was assessed across measures of *distress* and *quality of life*. *Distress* was measured using the Depression, Anxiety, Stress-21 scale [13], which provides subscale scores for depression, anxiety and stress, along with a total distress score. *Quality of Life* was assessed with differing measures in open access and the RCT. In open access, the briefer Medical Outcomes Study Short-Form-12 version 2 [SF-12v2; 14, 15] was selected to reduce respondent burden (i.e., 12 items instead of the RCT’s 30-item measure). This widely used and cancer-validated [16, 17] generic measure of health status provides summary scores of physical (physical component summary, PCS) and mental (mental component summary, MCS) health. Each domain is represented by six items, and is calculated and normalised according to published algorithms [14, 15]. Scores range from 0 to 100, with a mean of 50 and an SD of 10, with higher scores indicating better health [14, 15]. In the RCT, QOL was measured using the European Organisation for Research and Treatment of Cancer Quality of Life Core Questionnaire [EORTC QLQ C-30; 18], a 30-item assessment for cancer patients which yields a global QOL score, and five functional subscales (physical, emotional, social, role, cognitive). For the purposes of comparison with the two SF-12v2 domains in the present OA study, the EORTC QLQ C-30 *emotional functioning* and *physical functioning* subscales were selected.

### Statistical analyses

Descriptive statistics were conducted to summarise the sociodemographic and clinical characteristics of the sample, and to summarise the primary outcomes of uptake and engagement. For secondary outcomes, given the single group repeated measures design, planned analyses included repeated measures *t*-tests to compare the change over time from baseline to post-intervention in the OA group. However, given the small sample size post-intervention, inferential statistics were deemed inappropriate; thus, descriptive statistics (means, standard deviations) were conducted.

## Results

### Demographic and clinical characteristics

A total of 120 participants registered for the program. Table 1 depicts the demographic and clinical characteristics of OA participants (*n* = 120), with the baseline characteristics of the RCT intervention group (*n* = 78) provided as a reference. Of note, 42.5% of participants had missing data for the item ‘in what capacity are you using FMW’ as this was added mid-way through data collection. Of the remainder, most participants identified as a ‘person with cancer’ (43.4%), 5.8% were using the program as family members, and 7.5% were health care professionals. Participants were on average aged 55 years (SD = 17.2; range: 23–98), 78% were female, 71% identified as Australian ethnicity, 67% were partnered, 82% were tertiary educated, and 67% were employed. Most cancer participants had breast cancer (50%), with cancers of the prostate (4.2%), lung (4.2%), bowel (3.3%), melanoma (3.3%), and leukaemia (3.3%) also represented.

**Table 1:** Baseline demographic and clinical characteristics for OA vs RCT intervention samples

	Open access (*n* = 120)	RCT (*n* = 78)
Age	54.9 (17.2)	55.4 (11.1)
Female, *n* (%)	93 (77.5%)	65 (83.3%)
Married/partnered, *n* (%)	78 (67%)	65 (83.3%)
Employment status		
Employed Retired Unemployed	81 (67.5%) 22 (18.3%) 17 (14.2%)	45 (57.7) 23 (29.5%) 10 (12.8%)
Education		
Tertiary/TAFE High school Primary school	98 (81.7%) 14 (11.7%) 8 (6.7%)	55 (70.5%) 18 (23.1%) 5 (6.4%)
Ethnicity		
Australian Asian African Other Caucasian Other	85 (70.9%) 4 (3.3%) 1 (0.8%) 18 (15%) 8 (6.7%)	74 (94.9%) 1 (1.3%) 0 2 (2.6%) 1 (1.3%)
Annual income > $35,000	75 (62.5%)	55 (70.5%)
Cancer type		
Breast Melanoma Bowel Lymphoma Ovarian Prostate Lung Other*	60 (50%) 4 (3.3%) 5 (4.2%) 5 (4.2%) 2 (1.7%) 5 (4.2%) 5 (4.2%) 34 (28.3%)	52 (66.7%) 7 (9.0%) 8 (10.3%) 1 (1.3%) 1 (1.3%) 2 (2.6%) 0 (0%) 7 (9.0%)
Cancer stage		
0–2 3–4 Unclear Unknown Not applicable	57 (47.8) 44 (36.6) 0 7 (5.8%) 12 (10%)	30 (38.5%) 26 (33.3%) 15 (19.2%) 7 (9.0%) 0
Anticancer treatments received		
Surgery Chemotherapy Radiotherapy Other adjuvant treatment	79 (65.8%) 74 (61.7%) 56 (46.7%) 41 (34.2%)	70 (89.7%) 59 (75.6%) 43 (55.1%) 26 (33.3%)

*‘other’ = 28% (which includes: leukaemia (4); brain (1); anal (1); bladder/ureter (1); uterine and cervical (1); uterine/endometrial (1); carcinoid (2); cholangiocarcinoma (1); MM (1); clear cell renal carcinoma (1); metastatic breast (1); metastatic colon (1); GPA (1); mesothelioma (1); pancreatic (1); skin (1); testicular (1); thymoma (1))

### Uptake and engagement

**Fig. 1:**
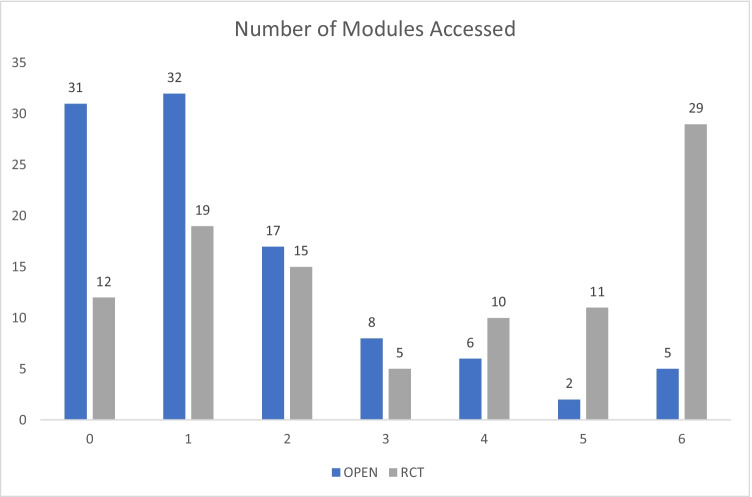
Comparison of usage between OA and RCT, as measured by the number of modules accessed (range = 0–6) by % of participants

Uptake during OA (*n* = 120) equated to 62.8% of uptake of the RCT (*n* = 191) in the matched recruitment timeframe. Overall, adherence was low, with an average of 1.5 modules completed per participant, and only 13% of participants completing an a priori defined therapeutic dose of 4 or more modules. More specifically, as Fig. 1 shows, 31% of OA users did not complete a single module (vs 12% in the RCT), 32% completed 1 module (vs 19% in the RCT), and only 5% of participants completed the full 6-module program (vs 29% in the RCT). In terms of which modules were most used, module 1 was the most frequently accessed—with 51% of participants exploring *diagnosis*/communicating with your team), and module 3 (emotional distress) being the second most accessed (40%). Thereafter, a linear drop in use occurred for modules 2, 4, 5, and 6.

### Effectiveness

Table 2 summarises the psychosocial outcomes for OA participants. Research attrition was high: of the 113 participants who completed baseline psychosocial measures, 13 completed post-intervention (11.5%). In comparison, 60/78 (76%) of RCT intervention participants completed post-treatment measures.

**Table 2:** Psychosocial outcomes

Outcome	Open access (*n* = 120)		RCT (*n* = 78)	
	Baseline (*n* = 113)	Post-treatment (*n* = 13)	Baseline (*n* = 78)	Post-treatment (*n* = 60)
Total distress	36.7 (26.5)	23.9 (20.7)	26.5 (21.7)	21.2 (21.1)
Depression	12.3 (10.0)	8.2 (8.3)	9.2 (9.2)	7.1(9.8)
Anxiety	9.4 (8.5)	5.7 (5.9)	6.6 (7.0)	4.9 (6.7)
Stress	15.0 (10.1)	10.0 (9.2)	10.7 (8.3)	9.2 (7.9)
QOL SF-12				
MCS	37.3 (12.63)	44.5 (12.1)	-	-
PCS	39.6 (11.0)	41.9 (11.1)	-	-
QOL EORTC				
EF	-	-	67.6 (22.3)	72.6 (23.3)
PF	-	-	80.4 (20.6)	78.4 (20.1)

*MCS* mental component summary (mental QOL), *PCS* physical component summary (physical QOL), *EF* emotional functioning (mental QOL), *PF* physical functioning (physical QOL)

#### Distress

Participants reported large decreases in distress from baseline (*M*_*pre*_ = 36.7; SD = 26.5) to post-intervention (*M*_*post*_ = 23.9; SD = 20.7). When looking at the DASS domains separately, there were consistent patterns of reductions from pre- to post-intervention in all three domains of depression, anxiety and stress (see Table 2).

#### QOL

In the OA phase, baseline scores in the PCS12 (*M*_*pre*_ = 39.6, SD = 11.0) and MCS12 (*M*_*pre*_ = 37.3, SD = 12.6) were over a standard deviation below the scoring normed average of 50. While only a 2-point average improvement from pre- to post-intervention was observed for the PCS12, a 7-point improvement in the MCS12 was observed over time. This pattern of findings was highly comparable to the EORTC QLQ-C30 EF and PF in the RCT.

## Discussion and conclusion

### Discussion

This analysis of uptake, engagement, and effectiveness during open access dissemination of an evidence based online program *Finding My Way* [1] has shown that — when compared to the original RCT — uptake and engagement was lower, but impact on outcomes remained. That is, there were sizeable changes in distress and mental QOL observed in OA, suggesting that — for those who use it — the program (even at low ‘dose’) was sufficient for improving wellbeing. However, these benefits must be balanced against the resource and sustainability considerations of continuing to maintain and host web-based resources when they are not used to their full or intended potential.

The key aim of the present study was to compare uptake, user characteristics, engagement, and outcome data with our earlier RCT findings; across all domains, several notable differences emerged. First, in terms of uptake, this open access study achieved 63% of the RCT sample size over a matched recruitment timeframe. There was no budget for advertising, nor additional staffing resources to the conduct this study; it was integrated within existing workloads; and recruitment was therefore not supported by the same measures usually employed in an RCT; this study did not have a dedicated research assistant available to screen potential participants, nor provide reminders to referring clinicians or to facilitate usage with participants. Instead, the present study relied purely on word of mouth and public forums to disseminate the program. Therefore, while the number of registered users in the present study was roughly a third lower, it is reassuring to know that even with passive recruitment strategies, uptake was maintained at modest rates. Future dissemination efforts need to focus on building the national and international profile of the program, and employ the strategies recommended by Davies et al. [9, 10] to increase referral rates, including ongoing HCP education/training and embedding programs within referral workflows.

Second, key differences emerged in the sociodemographic and medical profile of participants. While RCT and OA participants were comparable in age, more OA participants were men, unpartnered, ethnically diverse, tertiary educated, and employed than in the RCT. Medically, the sample was more heterogeneous in terms of cancer type in open access, with less participants having breast cancer than the RCT [1]. This is an important finding, and demonstrates that open access enables diversity that clinical research trials do not. This unique observation highlights the value of embedding open access dissemination into trial design, and we believe calls for future research efforts on how to disseminate open access more frequently.

Third, program engagement and research retention were much lower in OA than the RCT. Only 1.5 modules were completed on average in OA, and only 11.5% of participants elected to complete their post-intervention survey. While a much higher retention rate was found in the RCT, the post-treatment survey in open access was clearly worded as optional and only automated email reminders (i.e., no telephone reminders) occurred. While few open access studies have been reported in cancer, our engagement and retention findings are comparable to those observed by Davies et al. in their implementation of iCanAdapt, an online transdiagnostic clinician-monitored psychological therapy when offered as part of a clinical pathway for the management of early-stage cancer-related anxiety and depression [11]. After the successful completion of an earlier efficacy study [6]; their subsequent dissemination study found uptake and retention to be markedly lower, with only 25 patients self-referring, and of those only 44% of users completing at least 1 lesson [11]. Similarly, in an implementation study of an unguided sexual counselling intervention for cancer survivors, uptake was much lower, while attrition was double that seen in prior RCTs [19]. These findings are not unique to oncology, matching those of community digital mental health programs that have been disseminated for depression [20]. Collectively, these findings highlight a consistent profile of low/brief community-usage and high attrition of online programs, and speak to the need to rethink intervention design and engagement in future studies, as we discuss in detail below.

Finally, in terms of psychosocial outcomes, the present study found that OA users had (a) consistently and markedly higher baseline distress and impaired QOL and (b) larger changes from pre- to post-intervention, compared to their clinical trial counterparts. This is an important finding and again speaks to the importance of open access dissemination studies to evaluate intervention impact among more diverse and distressed populations. Our findings differed from those of Davies et al. [9]: when they implemented *iCanAdapt* as a component of a clinical pathway; the majority of self-referred participants were below the recommended distress threshold for using the program. However, we would argue that higher distress is somewhat to be expected, as RCTs employ strict eligibility criteria which may exclude those with advanced cancer and severe/psychiatric comorbidities, whereas in open access, while we sought to be clear who the program was designed for, anyone could (and did) sign up. Furthermore, motivations for signing up to the program likely differ from clinical trial (where altruism and wishing to contribute to science may drive consent in the absence of clinically significant distress) to open access (where distress is the main motivator for searching and signing up to programs, and where the possibility of control-group allocation may have previously deterred them from clinical trial registration) [19]. This tells us that open access is not simply an extension of an RCT and can augment the potential of an intervention by reaching those most in need — those who are more distressed, more diverse, and potentially more likely to be overlooked for clinical trial inclusion. It was promising to see that the current study largely replicated our RCT results in finding reduced distress and improved mental quality of life [1], particularly impressive in light of the low number of modules completed, and potentially suggests that you can get an effect with a modest dose. However, these psychosocial findings should be interpreted with caution, given the optional nature of the post-treatment survey and the resulting extreme post-treatment attrition. A response-bias may have occurred, and findings may not generalise to the broader oncology community. For example, it may be that only those who benefitted from the program or were doing well psychologically chose to complete the post-treatment survey. Furthermore, there may have been systematic differences in both baseline characteristics and program usage between the 13 participants who completed the post-treatment survey and those who did not. Given the small completer sample, it was not appropriate to conduct a sensitivity analysis.

#### Future directions

This study provides valuable insights into ‘real-world’ uptake, usage, and outcomes of digital psycho-oncology programs, which has important implications for two key areas of future research: evaluating methods of enhancing engagement, and testing the minimal engagement required to achieve effect. With respect to enhancing engagement, three options for this could include (1) *adding human interaction* via guidance, such as ‘coaches’; (2) *enhance the computer tailoring* of program content, such as by stratifying the content to the user’s cancer type, gender, age, sexual orientation, whether they have a partner or dependent-children, and (3) removal of *tunnelling* — to mimic traditional face-to-face therapeutic delivery, modules were released in a tunnelled format, once per week. However, adult learning literature [21] indicates that this is not how online users engage with content, and that having to ‘wait’ for desired content may be a disincentive to engagement. Addressing these three factors (in combination or in isolation) could result in a guided or highly curated version of the program, specifically matched to these characteristics, and would filter out obsolete/generic information [22]. Whether these new iterations improve engagement and retention could then be evaluated.

The second avenue for future research is that instead of focusing on how to *improve* engagement, one could focus on mapping intervention design around the engagement level we currently have. Indeed, recent studies suggest that it is not the amount of an intervention accessed that yields outcomes, but the self-selection of *activities of relevance* [23]. Even single-module use can lead to benefits in some studies [24, 25], while other studies show that the dose–response does not apply to digital health [26, 27]. Thus, instead of arbitrarily setting 4 modules as the benchmark of a ‘therapeutic dose’, and categorising participants as poor engagers if they complete less, future research could design *micro-interventions* for participants that only include a maximum of two modules, given that is the number of modules the data indicates users will complete. Micro-digital-health interventions are increasingly explored in other clinical populations, including body dissatisfaction [28] and mood [29–31]. Arising research questions would therefore be (a) how to maximise impact while minimising content and (b) can comparable outcomes be obtained through providing less, when compared to existing longer interventions.

### Practice implications

While digital mental health interventions are often hailed as a cost-effective method of delivering care, there are still a range of resource costs incurred including annual website hosting and maintenance costs, and time/staffing costs to address content updates. The present findings could therefore raise sustainability concerns, that the costs incurred outweigh the benefits provided. However, our group would argue that these concerns are premature, given that of the multiple indicators of impact, only user-engagement was potentially concerning, whereas uptake and psychosocial outcomes were promising. Indeed, in the current COVID-19 climate where access to traditional face-to-face care has been further restricted [32–35], digital psycho-oncology programs form an essential pathway for care provision — whether this be in a blended therapy format, delivered via telehealth with mental health professional guidance and oversight [36], or self-administered.

### Conclusion

Despite the limitations discussed, this open access study provides important data on the profile, usage, and outcomes achieved by community users of our digital psycho-oncology program *Finding My Way.* Findings that OA users were more medically and sociodemographically diverse and more distressed at baseline than their RCT counterparts, and — despite having lower usage of the program — achieved larger changes from baseline to post-program, will help to shape the way that future interventions are designed, tailored, and disseminated.

## Data Availability

Data is available from the corresponding author, upon request.
